# Tick-borne encephalitis vaccine breakthrough infections induce aberrant T cell and antibody responses to non-structural proteins

**DOI:** 10.1038/s41541-024-00936-7

**Published:** 2024-08-07

**Authors:** Amare Aregay, Jan Slunečko, Miša Korva, Petra Bogovic, Katarina Resman Rus, Nataša Knap, Jana Beicht, Mareike Kubinski, Giulietta Saletti, Tatjana Avšič-Županc, Imke Steffen, Franc Strle, Albert D. M. E. Osterhaus, Guus F. Rimmelzwaan

**Affiliations:** 1 Research Center for Emerging Infections and Zoonoses (RIZ), University of Veterinary Medicine Hannover, Foundation, Hannover, Germany; 2 Institute for Microbiology and Immunology, Faculty of Medicine, University of Ljubljana, Ljubljana, Slovenia; 3 Department of Infectious Diseases, University Medical Centre Ljubljana, Ljubljana, Slovenia; 4 Institute of Biochemistry, University of Veterinary Medicine Hannover, Foundation, Hannover, Germany

**Keywords:** Viral infection, Vaccines

## Abstract

Tick-borne encephalitis virus (TBEV) vaccine breakthrough (VBT) infections are not uncommon in endemic areas. The clinical and immunological outcomes have been poorly investigated. We assessed the magnitude and specificity of virus-specific antibody and T cell responses after TBE in previously vaccinated subjects and compared the results with those of unvaccinated TBE patients and study subjects that received vaccination without VBT infection. Symptomatic TBEV infection of unvaccinated study subjects induced virus-specific antibody responses to the E protein and non-structural protein 1 (NS1) as well as T cell responses to structural and other non-structural (NS) proteins. After VBT infections, significantly impaired NS1-specific antibody responses were observed, while the virus-specific T cell responses to the NS proteins were relatively strong. VBT infection caused predominantly moderate to severe disease during hospitalization. The level of TBEV EDIII- and NS1-specific antibodies in unvaccinated convalescent patients inversely correlated with TBE severity and neurological symptoms early after infection.

## Introduction

Tick-borne encephalitis virus (TBEV) infections can cause mild to severe neurological disease in endemic regions in Europe and Asia^1^. The virus is transmitted predominantly through tick-bites and belongs to the *Orthoflavivirus* genus of the family *Flaviviridae*^2,3^. In the absence of effective antiviral therapy, vaccination is the main measure to prevent TBE. Two licensed inactivated TBE vaccines (FSME-IMMUN® and Encepur®) are available in Europe. For these vaccines to be effective, repeated doses are required, and depending on the age and immune status of the vaccinees often booster vaccinations are needed to maintain protective immunity^3,4^. Despite the availability of effective vaccines, up to 15,000 clinical cases are reported worldwide annually^5^. A proportion of these cases can be attributed to subjects that were vaccinated and the majority of these so-called vaccine breakthrough (VBT) cases have a more severe course of disease^6–9^. The incidence of TBE in endemic areas is increasing and the geographical spread of TBEV infection is expanding^1,10,11^.

Like other Orthoflaviviruses, the TBEV genome encodes 3 structural proteins (envelope (E), (pre)membrane (prM), and capsid (C)) and 7 non-structural proteins (NS1, NS2A, NS2B, NS3, NS4A, NS4B, and NS5)^12^. The E protein is the major target for virus neutralizing (VN) antibodies and the presence of serum E-specific IgM and IgG antibodies is used for diagnostic purposes and to assess virus-specific immunity induced after infection or vaccination^13^. In particular, antibodies to domain III of the E protein (EDIII) have VN activity and contribute to protective immunity^14^. The viral non-structural protein 1 (NS1) is involved in viral replication but also induces virus-specific antibody and T-cell responses, which contribute to protective immunity^12,15^. Furthermore, the presence of antibodies to the NS1 protein, which is also secreted from infected cells as a hexamer, potentially allows the differentiation of infection- and vaccine-induced immune responses^16,17^. Vaccination often induces lower TBEV-specific neutralizing antibody responses than natural infection^16,17^ while VBT infections induce strong VN IgG responses, suggestive of anamnestic responses^18–20^. Whether the overt clinical symptoms often seen in VBT infections correlate with anamnestic antibody responses, intrathecal inflammation^21,22^ or other virulence-associated factors is largely unknown.

Most studies on the induction of TBEV-specific immunity focused on virus-specific antibody responses in hospitalized patients shortly after infection. Knowledge on virus-specific T-cell responses is sparse and available for only a limited number of epitopes^23–25^. Severe TBE may have long-term post-encephalitic sequelae including lasting motor deficit, cognitive and speech impairment^26^. In the present study, we addressed two research questions. First, we investigated if TBEV-specific antibody and T cell levels in convalescent samples correlated with disease severity during the acute TBE illness and the presence of neurological sequelae at a later stage. Second, we investigated the impact of prior vaccination on the immunological and clinical outcome of infection in VBT cases. To this end, the magnitude and specificity of antibody and T cell responses were assessed in samples obtained from 59 unvaccinated convalescent TBE patients and 10 VBT cases, of which clinical records during infection and long-term follow-up were known. Subjects that received vaccination only and unvaccinated unexposed subjects were included as controls. The magnitude of the antibody response to EDIII and NS1 inversely correlated with disease severity during hospitalization at the time of acute illness. Compared to unvaccinated TBE patients, the VBT cases displayed more potent T cell responses to NS proteins, but impaired antibody responses to the NS1 protein. The data are discussed in the light of vaccine effectiveness and immune monitoring of vaccinated subjects to achieve optimal protection against TBEV infections.

## Materials and methods

### Study subjects

Ninety-three study subjects were enrolled in the study. Fifty-nine were TBE patients not vaccinated against TBEV or other Orthoflaviviruses (unvaccinated TBE patients (unvaccinated Pts.)). Ten were TBE patients that had been vaccinated against TBE (2, 3, or >3 doses of TBEV vaccines given 1 month–10 years before infection), so-called VBT cases. Seventeen study subjects had not been infected but received 2 or 3 doses of TBEV vaccines (FSME-IMMUN® (*n* = 15) or Encepur® (*n* = 2) (vaccinees)). Seven TBEV seronegative subjects were not vaccinated or infected (unexposed). All TBE patients were seen at the University Medical Centre Ljubljana, Slovenia, and blood samples were collected 1–17.4 years (1–16.3 years (median = 6.3 years) for VBTs and 1.3–17.1 years (median = 4.5 years) for unvaccinated patients) after initial diagnoses (Table 1). The group of study subjects that received vaccination only were recruited in Hannover, Germany, and blood samples were collected 0.1–16 years after the last vaccination (Table 1). TBEV seronegative unexposed subjects were also recruited in Hannover, Germany. The presence of serum antibodies was confirmed by TBEV IgG/IgM ELISA (Anti-TBE Virus ELISA IgG/IgM Kit, Euroimmun). Individuals that received vaccination against Orthoflaviviruses other than TBEV were not included in the study.

**Table 1:** Clinical profile and socio-demographic characteristics of study subjects

	Unexposed (*n* = 7)	Vaccinees (*n* = 17)	Unvaccinated patients (*n* = 59)	VBT cases (*n* = 10)
Age (years); Median (range)	-	30 (24–71)	64 (23–83)	64 (33–76)
Gender (F)	-	13	26	2
Duration (years from diagnosis to sample collection); median (range)	-	1.36 (0.1–16)	4.5 (1.3–17.4)	6.25 (1–16.5)
Previous vaccination for TBE	-	NA	-	10 (1 month–10 years prior to infection)
Previous vaccination for JEV or YF	-	-	-	-
Immunosuppression at the time of sampling	-	-	2	-
Blood leukocyte count (×10^9^ cells/L); median (range)	NA	NA	9.8 (3.8–25.0) information for 56 pts	10.4 (5.7–18.5) information for 8 pts
Serum CRP level (mg/L); median (range)	NA	NA	7.5 (<3–114) information for 56 pts	8.5 (<3–56) information for 8 pts
CSF findings Leukocyte count (×10^6^/L); median (range) Protein level (g/L); median (range)	NA	NA	102 (6–725) information for 59 pts 0.81 (0.27–2.57) information for 57 pts	138.5 (21–853) information for 10 pts 1.16 (0.52–1.60) information for 8 pts
Clinical manifestation				
Meningitis (M), *n* (%)	NA	NA	20 (33.9%)	1 (10%)
Meningoencephalitis (ME), *n* (%)	NA	NA	30 (50.8%)	7 (70%)
Meningoencephalomyelitis (MEM), *n* (%)	NA	NA	9 (15.3%)	2 (20%)
Severity score			(available for 54/ 59 patients)	(available for 8/ 10 patients)
Mild (0–8)	NA	NA	19^a^	0
Moderate (9–22)	NA	NA	21^b^	6^d^
Severe (>22)	NA	NA	14^c^	2^e^
Outcome (on the date of sample collection)				
Favorable	NA	NA	38	7
Subjective symptoms affecting quality of life	NA	NA	14	3
Objective neurological sequelae	NA	NA	7	0

^a^All had meningitis.

^b^19 had ME and 2 had MEM.

^c^8 had ME and 6 had MEM.

^d^All had ME.

^e^all had MEM.

Clinical manifestation and disease severity of TBE patients were defined as described previously^27^. All patients included in this study had neurological symptoms, cerebrospinal fluid pleocytosis and were categorized based on clinical manifestations of meningitis, meningoencephalitis or meningoencephalomyelitis during hospitalization (Table 1). Furthermore, the disease severity of patients was quantitatively assessed by means of standardized questionnaire for 62/69 (90%) of patients. The individual scores given to specific signs and symptoms of TBE were tallied to give a clinical score (0–8 = mild, 9–22 = moderate, and >22 = severe), as described previously^27^ (Table 1). The long-term disease outcome at the time of sample collection was also assessed based on the subjective or objective TBE-associated symptoms/signs, as described previously^26^ (Table 1).

### Ethics statement

This study was approved by the national medical ethics committee of the Republic of Slovenia (No 152/06/12 and No 0120-467/2017/3) and by the local ethical committee of Hannover Medical School (MHH) (Permit number 3393–2016). All patients provided written informed consent for participation in this study.

### Collection of serum and peripheral blood mononuclear cell (PBMC) samples

Serum was isolated from blood samples collected from each TBE patient by centrifugation at 600 × *g* for 10 min, or for vaccinated study subjects and unexposed individuals at 1800 × *g* for 15 min, aliquoted and stored at −80 °C until further use. PBMCs were isolated from blood treated with ethylenediamine-tetra-acetic acid (EDTA; 1.8 mg/ml of blood) by Ficoll-Paque PLUS (GE Healthcare, Uppsala, Sweden) or Lymphoprep (Stem cell Technologies, (for vaccinees and unexposed)) density gradient centrifugation as described previously^22^. Isolated PBMCs were resuspended in freezing medium containing RPMI-1640 (10%) + fetal bovine serum (FBS; 80%) + dimethylsulfoxide (DMSO; 10%), aliquoted and cryopreserved until further use.

### Synthetic peptides

Twelve peptide pools (C_1-117_, E_1-255_, E_245-496_, NS1_1-183_, NS1_173-352_, NS3_1-215_, NS3_205-419_, NS3_409-621_, NS5_1-231_, NS5_221-459_, NS5_449-683_, NS5_673-903_) comprising 15-mer synthetic peptides that overlap by 11 amino acids (≥75% purity, GenScript Biotech Corp, Piscataway Township, New Jersey, USA) and spanning the C, E, NS1, NS3 and NS5 proteins of TBEV strain Neudoerfl (European subtype, UniProtKB: P14336) were prepared. These proteins were selected because they constitute the major targets for cellular immune responses to TBEV^25,28,29^. Depending on the size of proteins, up to four peptide pools per protein were prepared by reconstituting the lyophilized peptides in 100% DMSO (Hybri-Max™, Sigma–Aldrich, St. Louis, Missouri, USA) to yield a stock concentration of 0.2 mg/ml per peptide within each pool.

### Interferon-gamma (IFN-γ) enzyme-linked immunosorbent spot (ELISpot)

PBMCs (2.5 × 10^5^ cells/well in duplicate) were restimulated with each of the 12 peptide pools described above at a final concentration of 1 µg/ml. Also 0.5% DMSO in culture medium (similar to the DMSO concentration for peptide pools) or anti-CD3 (Mabtech, 1:1000 with 2.5 × 10^4^ PBMCs) were included in each assay as negative and positive controls, respectively. Subsequently, re-stimulated PBMCs were transferred to washed and RPMI-1640 containing 10% FBS (R10F) conditioned ELISpot plates and incubated for 20 h at 37 °C and 5% CO_2_. Plates were developed according to manufacturer’s instructions (Mabtech, Nacka Strand, Sweden) and spots were counted using ImmunoSpot® S6 Ultimate Reader fitted with ImmunoSpot Software (Cellular Technology Limited). Results were calculated and expressed as spot-forming units (SFU) per 1 × 10^6^ PBMCs after subtraction of mean values of negative control (DMSO) stimulations. A cut-off value for a positive response was determined based on the average value from DMSO negative controls of all study subjects plus two standard deviations. A response with >10 SFU/1 × 10^6^ PBMCs was considered positive.

### IgG/IgM enzyme-linked immunosorbent assay (ELISA)

A commercially available ELISA kit (Anti-TBE Virus ELISA IgG/IgM Kit, Euroimmun) was used for the detection of TBEV-specific IgG and IgM antibodies in serum according to the manufacturer’s instructions^30^.

### Multiparametric flow cytometry analysis

Between 1.5 × 10^6^ and 2 × 10^6^ PBMCs were stimulated with peptide pool(s) that gave a response in the IFN-γ ELISpot, always maintaining a DMSO concentration of <1%. A mixture of anti-CD3 (0.1 µg/ml), anti-CD28 (1 µg/ml), and anti-CD49d (1 µg/ml) (BD Biosciences) stimulation or DMSO stimulation served as positive and negative controls, respectively. Stimulated PBMCs were incubated for 20 hours at 37 °C, 5% CO_2_ with Brefeldin A (7 µg/ml; Sigma–Aldrich) added during the last 4 hours to block cytokine secretion. Subsequently, cells were washed and stained with LIVE/DEAD™ Fixable Near-IR Dead Cell Staining kit to exclude dead cells and surface staining for CD3, CD4, CD8, CD14, CD19, CCR7, CD45RA, CD29, and CD49D with anti-human fluorochrome-labeled monoclonal antibodies was performed (Supplementary Table 1). Intracellular cytokine staining for interferon-γ (IFN-γ), interleukin-2 (IL-2), and tumor necrosis factor-α (TNF-α) was performed after fixation and permeabilization using Cytofix/Cytoperm solution (BD Biosciences) according to manufacturer’s instructions. Samples were acquired using LSR Fortessa X-20 flow cytometer (BD Biosciences) using BD FACS Diva software (version 9.0, BD Biosciences). Data were analyzed using FlowJo v.10.8.1 (FlowJo LLC) software. Naïve T cells (defined as CCR7^+^CD45RA^+^) were excluded from all cytokine analyses and values for cytokines responses were provided after subtraction of background response from DMSO controls. A value above 0.01 was considered positive. The gating strategy and representative plots are provided in Supplementary Fig. 2.

### Virus-neutralization test (VNT)

Heat-inactivated sera (30 min, 56 °C) from all patients and vaccinees were two-fold serially diluted (in triplicate, starting at 1:20 dilution) in A549 infection medium. Sera from TBEV naïve donor and known TBEV high responder were included as negative and positive controls, respectively^31,32^. Diluted sera were incubated with 100 TCID_50_ TBEV strain Neudoerfl for 1 h at 37 °C and 5% CO_2_ and the mixture transferred to confluent A549 cells (at least 80%) and incubated for further 6 days at 37 °C and 5% CO_2._ The reciprocal of the highest serum dilutions without visible cytopathic effect (CPE) was used to calculate the VN titer. Correct TCID_50_ of input virus was confirmed through back-titration. A VN titer >20 was considered positive.

### Luciferase immunoprecipitation system (LIPS) assay

Mammalian expression plasmid pcDNA3.1/Zeo expressing TBEV proteins (EDIII or NS1) fused to Nano luciferase (NLuc) or secreted NLuc (secNLuc) alone were transfected to Cos-1 cells and supernatants containing the fusion proteins or secNLuc alone were harvested, normalized to 1 × 10^6^ relative light units (RLU) and incubated with heat-inactivated sera at 1:100 dilution. The assay detects both IgG and IgM antibodies and was performed as described previously^33^. Additional incubation with protein A beads (Thermo Fischer) on filter plates (MultiScreenHTS BV Filter Plate, Millipore) allowed the pull-down of bound fusion protein antigens. The luminescence was measured with microplate reader infinite 200Pro (Tecan) with Tecan i-control software (version 2.0.10.0, Tecan). Sera from TBEV non-exposed individuals and sera with known high titers for EDIII and NS1 were included in the assay to serve as negative and positive controls, respectively. The assay was performed in triplicates, mean of triplicate values was calculated, and data was expressed as RLU. A cut-off RLU for positive response was determined based on the mean value of negative control samples incubated with secNLuc alone. A value higher than the average of negative controls plus three times the standard deviation was considered positive.

### Statistical analyses

GraphPad prism V9 (GraphPad Software, LA Jolla California) software was used for Statistical analyses of data. D’Agostino & Pearson test and Kolmogorov–Smirnov test were used to determine the statistical distribution of samples. A two-tailed Spearman correlation test was employed to calculate r and *p* values in the correlation analyses. For the comparison of groups, one-way or two-way analyses of variance (ANOVA) or two-tailed Kruskal–Wallis test with multiple comparison tests were performed for samples with or without normal distribution, respectively. The statistical test used for each graph is indicated in the legends. *P < 0.05, **P < 0.01, ***P < 0.001, ****P < 0.0001.

## Results

### TBEV VBT infections cause predominantly moderate to severe TBE

**Fig. 1:**
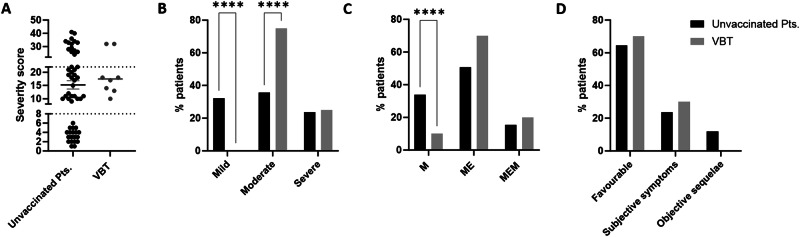
TBE severity at the time of acute illness in TBE and VBT TBE cases. **A** Disease severity of vaccinated and unvaccinated TBE patients based on severity score of TBE during hospitalization. Dashed lines indicate cut-off scores for categorizing mild (0–8), moderate (9–22), and severe (>22) infections. **B** Proportion of unvaccinated patients and vaccine breakthrough (VBT) cases with mild, moderate, and severe disease during hospitalization. **C** Proportion of TBE patients according to basic neurological manifestations shortly after infection (M meningitis, ME meningoencephalitis, MEM meningoencephalomyelitis). **D** Proportion of TBE patients according to long-term clinical outcome by the time of convalescence. Light gray = VBT, Black = Unvaccinated patients. Two-way Analyses Of Variance (ANOVA) with multiple comparison test was performed for comparison of groups. ****p < 0.0001.

None of the patients with VBT infection of which clinical scores were available presented with mild disease but suffered from moderate (75%) and severe (25%) TBE based on the severity score (Fig. 1A, B). In contrast, 19 out of 54 (35%) unvaccinated TBE patients had mild TBE (Fig. 1A, B). The severity score coincided with the clinical presentation. Only one of the VBT cases displayed meningitis (10%) while the remaining patients suffered from meningoencephalitis (70%) or meningoencephalomyelitis (20%) during hospitalization (Fig. 1C). For the unvaccinated patients the proportion of cases with meningitis, meningoencephalitis, and meningoencephalomyelitis were 33.9%, 50.8%, and 15.3%, respectively (Fig. 1C). VBT cases had favorable long-term outcome (70%) or subjective symptoms affecting quality of life (30%), but no objective neurological sequelae (Fig. 1D). Unvaccinated patients had 64.4% favorable outcome, 23.7% subjective symptoms and 11.9% objective neurological sequelae (Fig. 1D).

### Differential TBEV-specific antibody responses following vaccination, infection, or VBT infection

**Fig. 2:**
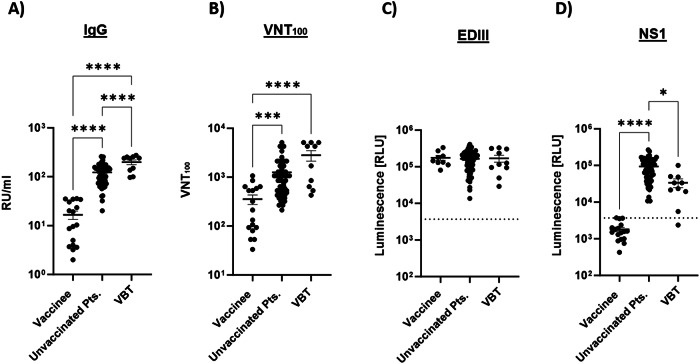
Differential TBEV-specific antibody responses following vaccination, TBE or VBT TBE. **A** TBEV-specific IgG titer in serum samples obtained after vaccination, infection or VBT infection. **B** Vaccination-, infection- or VBT infection-induced virus neutralizing titers (VNT_100_). **C**, **D** TBEV EDIII- and NS1-specific serum antibodies in vaccinees, unvaccinated patients, and VBTs. Horizontal lines indicate the mean with standard errors of the mean (SEM). Dashed lines indicate cut-off values for positive responses as detailed in the methods section. Two-tailed Kruskal–Wallis test or One-way ANOVA with multiple comparison test was performed for comparisons of groups. *p < 0.05, ***p < 0.001, ****p < 0.0001.

TBEV-specific IgG and VN antibodies were induced after TBEV vaccination, but the levels were significantly lower than after infection or VBT infection (Fig. 2A, B). Interestingly, the virus-specific IgG titer was significantly higher in VBT cases than in the TBE patients who did not receive vaccination (Fig. 2A). Also, the antibody responses to TBEV EDIII and NS1 proteins were investigated (Fig. 2C, D). As expected, vaccination induced EDIII-specific but not NS1-specific antibodies (Fig. 2C, D). Remarkably, the NS1-specific antibody levels were significantly lower in the VBT cases than in TBE patients, who had not been vaccinated (Fig. 2D). In contrast, the antibody titers to the EDIII domain did not differ between these two groups (Fig. 2C). Collectively, natural infections induce stronger antibody responses than vaccination. Furthermore, strong antibody responses were observed in the VBT cases, however, the response to the NS1 protein was significantly lower.

### TBEV EDIII- and NS1-specific antibody titers years after acute illness correlate with mild acute disease

We also investigated to what extent disease severity shortly after infection influenced the long-term immunological outcome of infection. Unvaccinated patients with mild TBE maintained significantly higher titers of TBEV EDIII- or NS1-specific antibodies than patients who suffered from more severe disease (Supplementary Fig. 1A, B). Likewise, patients with meningoencephalomyelitis, the most severe clinical manifestation of TBE, had relatively lower TBEV EDIII-specific antibody titers than patients who had meningitis only (Supplementary Fig. 1A). These disease severity-associated differences were not observed for virus-specific IgG antibody and VN antibody titers (Supplementary Fig. 1C, D). We also compared the antibody levels in the convalescent samples with the long-term outcome of the TBEV infections. Relatively low TBEV-specific IgG, EDIII, and NS1 antibody levels were observed in patients with objective neurological sequelae, although the difference with patients that had a more favorable disease outcome was not statistically significant (Supplementary Fig. 1A–C).

### Differential T cell responses to non-structural proteins in VBT cases and unvaccinated TBE patients

**Fig. 3:**
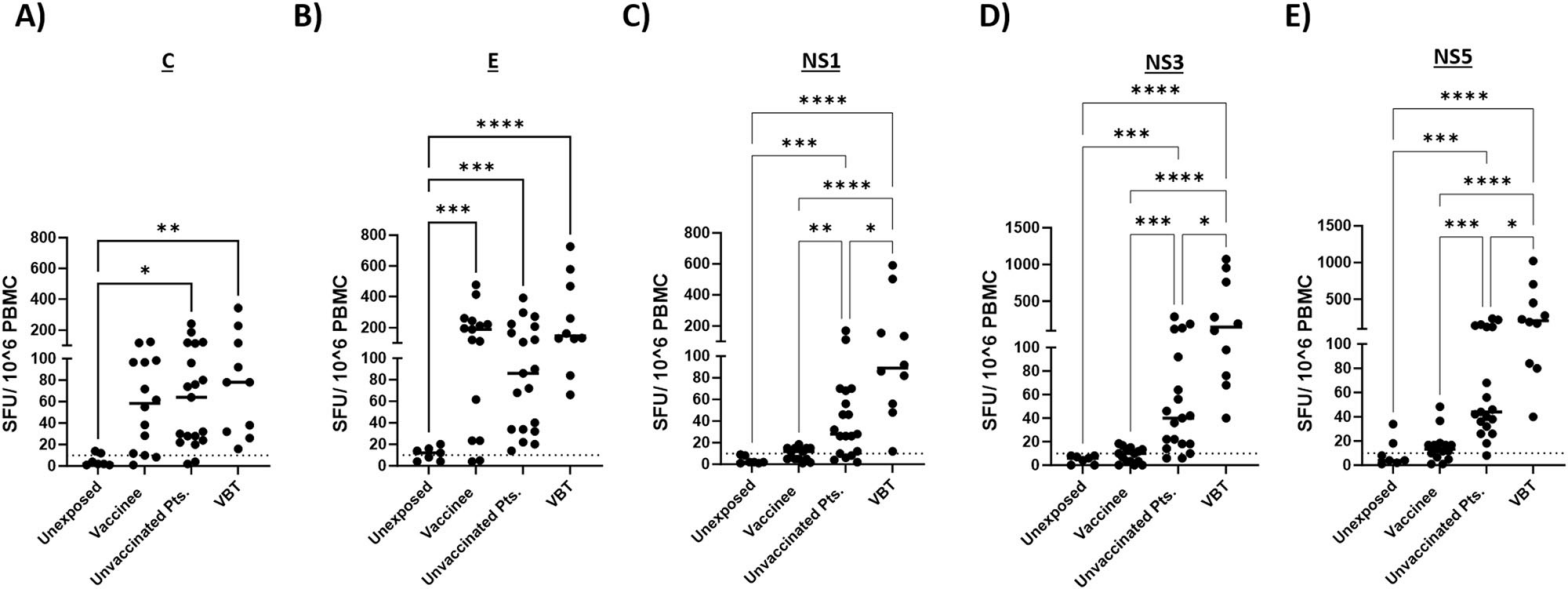
Differential T cell responses to non-structural proteins in VBT cases and unvaccinated TBE patients. **A**–**E** TBEV-specific IFN-γ spot forming units (SFU) per 1 × 10^6^ PBMC after stimulation with peptide pools derived from structural (**A, B**) and non-structural (**C**–**E**) TBEV proteins after vaccination, TBE, VBT TBE or unexposed subjects. The sum of individual values obtained with peptide pools per TBEV protein was used to calculate the response to each protein. Each dot represents single study participant and the horizontal lines indicate median values. Dashed lines indicate cut-off values for positive responses as detailed in the methods section. Two-tailed Kruskal–Wallis test or One-way ANOVA with multiple comparison tests was performed for comparisons of groups. *p < 0.05, **p < 0.01, ***p < 0.001, ****p < 0.0001.

**Fig. 4:**
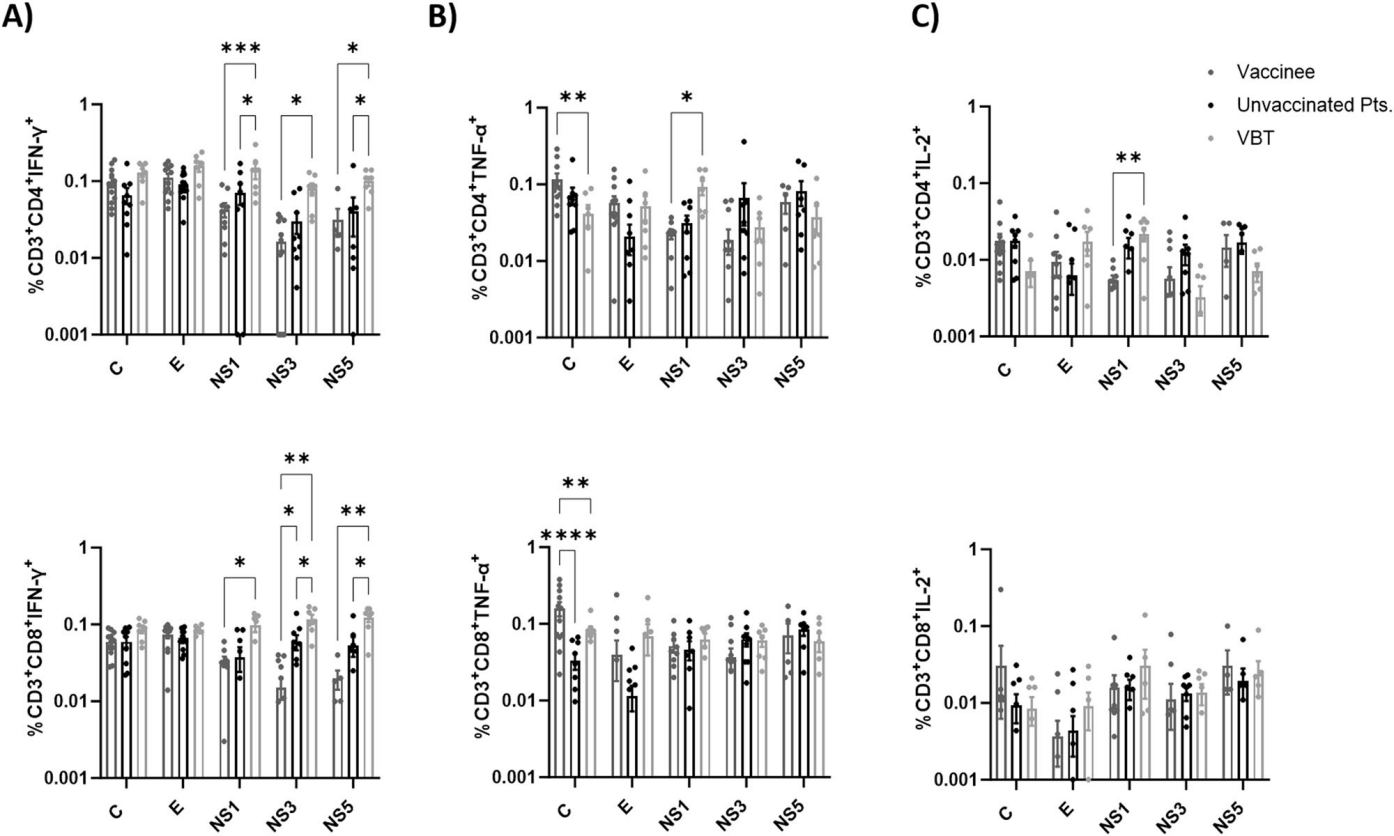
Flow cytometry analysis of TBEV-specific CD4^+^ and CD8^+^ T cells after vaccination, infection, and VBT infection. **A** Frequencies of CD4^+^ (top) or CD8^+^ (bottom) T cells producing IFN-γ against the structural or non-structural TBEV proteins following vaccination, infection, and VBT infection. The respective CD4^+^ (top) or CD8^+^ (bottom) T cells producing TNF-α (**B**) or IL-2 (**C**) in response to the investigated TBEV proteins are depicted. Production of each cytokine was analyzed after exclusion of naïve T cells in both CD4^+^ and CD8^+^ T cell subsets. A response above 0.01% was considered positive. Each dot represents single study participant and horizontal lines indicate mean with SEM. Two-way ANOVA with multiple comparison tests was performed for comparison of groups. *p < 0.05, **p < 0.01, ***p < 0.001, ****p < *0.0001*.

Upon vaccination, TBEV infection and in the VBT cases T cell responses towards the structural (E and C) proteins were readily detected by IFN-γ ELISpot assay. The magnitude of these responses did not differ significantly between the three groups (Fig. 3A, B). In contrast, and as expected, the study subjects that received TBE vaccine only displayed very low reactivity to the non-structural proteins NS1, NS3, and NS5 (Fig. 3C–E). In TBE subjects, T cell responses to the non-structural proteins were detected in the majority of these patients. As expected, none of the structural or NS proteins gave rise to detectable IFN-γ response in unexposed subjects (Fig. 3A–E). Of special interest, the magnitude of the T cell responses to the NS proteins was higher in the VBT cases compared to unvaccinated TBE patients (Fig. 3C–E). Intracellular cytokine staining and flow cytometry showed that the TBEV-specific T cell detected by IFN-γ ELISpot assay were both CD4 and CD8 positive (Fig. 4A). The frequency of CD4^+^/IFN-γ^+^ T cells directed to NS1 and NS5 and the frequencies of CD8^+^ /IFN-γ^+^ T cells to NS3 and NS5 were significantly higher in VBT cases than in unvaccinated TBE cases, confirming the results obtained in the IFN-γ ELISpot assay (Fig. 4A and Supplementary Fig. 3). Vaccination with inactivated vaccine also gave rise to TNF-α^+^ CD4^+^ and CD8^+^ T cell responses to the C protein, which were higher than after TBEV infection (Fig. 4B). Relatively high frequencies of TNF-α^+^ and IL-2^+^ CD4^+^ T cells to the NS1 protein were detected in VBT cases (Fig. 4B, C). Collectively, TBEV vaccination induced virus-specific T cell responses directed to the structural proteins C and E, whereas TBEV infection also induced T cell responses to the NS proteins. In general, the responses to the NS proteins were stronger in the VBT cases than in the unvaccinated TBE patients, in particular the response to NS1.

The disease manifestation early after TBEV infection did not seem to influence the long-term magnitude and specificity of the T cell responses (Supplementary Fig. 3A–E), although the T cell response to the C, NS1, and NS3 proteins in unvaccinated TBE patients with meningoencephalomyelitis seemed to be lower than patients with meningitis (Supplementary Fig. 3A, C, D). Unvaccinated patients who had long-term objective neurological sequelae had lower E-specific T-cell response compared to patients with favorable outcome (Supplementary Fig. 3G).

## Discussion

In the present study, we analyzed the magnitude and specificity of the TBEV-specific antibody and T-cell responses in convalescent samples obtained from three study cohorts. (1) Subjects that received one of the commercially available TBEV vaccines, (2) previously hospitalized TBE patients, and (3) previously hospitalized patients that received vaccination prior to contracting TBE. Furthermore, the TBEV-specific immune responses in these three groups were evaluated in light of the severity of the disease shortly after infection as well as after the development of long-term sequelae, and the effects of TBEV vaccination were discussed based on the findings.

Vaccination is an important preventive measure against TBE and the most important correlate of vaccine-induced protection are VN antibodies, mainly directed against the E protein^13,17^. It is recommended to offer booster vaccination every 3–10 years, to retain virus neutralizing antibody levels at protective levels^34–36^. Indeed, the subjects in the present study, who only received their primary round of vaccinations, had relatively low VN and virus-specific IgG antibody titers compared to the TBE patients and the VBT cases, which is in concordance with the results of other studies^14,16,17^. Vaccination did not induce detectable NS1-specific antibodies, most likely because NS1 is not a major component of inactivated virus particles, the basis of the inactivated TBE vaccines currently in use^1,17^. Other studies have reported the induction of NS1-specific antibodies with these TBEV vaccines^37,38^. The time point of serum sampling may be at the basis of this discrepancy and these findings suggest that TBEV vaccines still contain trace amounts of NS1. Of note, immunity to NS1 has been shown to afford partial protection against TBEV and is most likely mediated by NS1-specific antibodies^15^. Although vaccine effectiveness is high, VBT infections are reported often with an unfavorable course of the disease^6,9,18,39^. Also, the VBT cases included in our study suffered from moderate to severe disease during the early stages of TBE, with a higher likelihood to develop severe neurological manifestations. Although the VBT cases were randomly selected based on the availability of serum samples and corresponding PBMCs, we cannot exclude that we might have missed mild cases because of the small sample size.

Upon TBEV infection, durable virus-specific antibody response were induced as detected by ELISA, VN assay, and LIPS assays. Not only (VN) antibodies to the E proteins were detected but also antibodies to NS1 (Fig. 2). Of special interest, symptomatic infection in vaccinated subjects induced even stronger IgG antibody responses as measured by ELISA, suggestive of anamnestic antibody responses. In the VBT cases, the convalescent NS1-specific antibody levels were significantly lower than in the unvaccinated TBE patients. The low levels of antibodies to NS1 detected in the VBT cases may seem paradoxal. However, oligomeric forms of NS1 are also secreted from infected cells in large quantities. Of note, NS1 is also known to be a virulence factor that can affect the integrity of endothelial cells leading to vascular leakage or breakdown of the blood–brain barrier and disease severity correlated with serum NS1 levels^40,41^. The presence of NS1 in serum may have caused selective depletion of NS1-specific B cells and/or absorption of NS1-specific antibodies and antibody-NS1 complexes may have been removed from circulation by antigen trapping in secondary lymphoid organs^42^. However, the differential kinetics of NS1 secretion during viremia and the subsequent antibody responses makes this explanation unlikely. Alternatively, yet unexplained factors may underlie poor maintenance of protective antibody levels in VBT cases after vaccination rendering these subjects susceptible to infection and disease severity-associated decline of protective antibody levels. Of interest, patients that experienced severe disease shortly after infection had the lowest EDIII- and NS1-specific antibody levels years later indicating that the induction of these antibodies afforded some protection against developing severe TBE. Similar findings have been obtained in TBE animal models and for other Orthoflaviviruses^15,41,43–46^.

In all three groups, (memory) T cells to the structural C and E proteins were detected by IFN- γ ELISpot. As expected, T cells to the non-structural proteins NS1, NS3, and NS5 were virtually absent in the study subjects that received the vaccine only. In the TBEV-infected individuals, T cell responses to the NS proteins were readily detectable but the frequencies of NS protein-specific T cells were significantly higher in the VBT cases than in patients with TBE who had not been vaccinated. These results were confirmed by intracellular IFN-γ staining and flow cytometry, which indicated that virus-specific T cells were both CD4^+^ and CD8^+^. The differential immunological outcome of infection between VBT cases and unvaccinated TBE patients is intriguing. We speculate that in the VBT cases, the vaccine-induced antibody levels had dropped to unprotective levels. The remaining antibodies may even have caused antibody-dependent enhancement of infection (ADE), a phenomenon that is well-known for other members of the Orthoflaviviruses. ADE may also explain the predominantly moderate to severe infections amongst the VBT cases and may have been associated with increased virus replication and production of viral proteins, including the NS proteins. However, the discrepancy in kinetics between virus replication and viremia on the one hand and onset of clinical signs on the other, makes assessment of viral load almost impossible. We speculate that increased antigen loads may have induced more potent T cell responses to the NS proteins in the VBT cases compared to their non-vaccinated counterparts. Comparison of T cell responses to NS proteins, after stratifying for NS1-specific antibody levels between the two groups, showed that the higher NS-specific T cell responses in VBT cases were independent of the antibody levels to NS1 (Supplementary Fig. 5).

Other factors, such as intercurrent infections between clinical onset and sample collection, that could have influenced TBEV-specific antibody and T-cell responses, cannot be ruled out. Such infections with TBEV or other flaviviruses are only detected when they are symptomatic. Since the study was designed as a cross-sectional study, no additional serum or PBMC was collected between clinical onset and sample collection during the convalescent phase, to test e.g., for antibody titer rises. Furthermore, the relatively small number of VBTs and variability in immune responses precluded further stratified analyses that would have given even more insight on the correlation between clinical and immunological outcome of VBT infection. It should be considered that VBT cases constitute a small proportion of all TBE patients in the study area and eslewhere^18^, and in-depth immunological investigation of VBT TBE cases has not been done before.

Previously, we have shown that strong virus-specific T cell responses early after infection correlated with mild disease and favorable outcomes of infection in hospitalized TBE patients^47^. This correlation was not clear in the present study using PBMCs obtained during convalescence, although some patients with long-term neurological sequelae had poor T cell responses, in particular to the C and E proteins. At present, it is not clear if virus-specific T cells solely have protective effects or may also contribute to the pathogenesis of TBE. We (Supplemental Fig. 4) and others have shown that TBEV-specific CD8^+^ T cells may express alpha4 beta1 integrin (α4β1), which among others is a marker for homing to the central nervous system (CNS)^25^, where T cells may have detrimental pro-inflammatory effects^21,22^. In the present study, vaccine-induced TNF-α^+^ CD4^+^ and CD8^+^ T cell responses to the C protein were higher after vaccination than infection. Previous studies also showed that vaccine-induced CD4^+^ T cells specific for the structural proteins produced less IFN-γ but more TNF-α or IL-2 than CD4^+^ T cells induced shortly after TBEV infection^28,48^.

Collectively, we have examined and compared the virus-specific immunological outcome of TBEV vaccination and infection in previously hospitalized TBE patients that received prior vaccination or not. The comparative immune profiling of serum antibodies and T-cell responses in convalescent samples revealed striking differences between the three experimental groups. TBE vaccination induced appreciable VN antibody responses and virus-specific T cell responses to the structural proteins until the time point of samples collection. However, antibody responses induced by infection were generally stronger as was the case for infection-induced T cell responses, in particular to the NS proteins, which were absent after vaccination. Of particular interest were the differences in CD4^+^ and CD8^+^ T cell responses to the NS proteins and the antibody response to NS1 between VBT cases and unvaccinated TBE patients. We hypothesize that the strong T cell responses to NS protein observed in VBT cases were predisposed by prior TBE vaccination and subsequent ADE of infection. These findings warrant further investigation and underscore the need for booster vaccinations with the current inactivated virus vaccines to ensure that antibody levels are at protective levels at all times.

### Supplementary information

Supplementary Information

## Data Availability

All data generated or analyzed during this study are included in this published article [and its supplementary information files].
