# Supplementary material for: Tick-borne encephalitis vaccine breakthrough infections induce aberrant T cell and antibody responses to non-structural proteins
Source: NPJ Vaccines. 2024 Aug 7;9:141. doi: 10.1038/s41541-024-00936-7 (PMC11306791; doi:10.1038/s41541-024-00936-7)
Supplement: Supplementary file 1 — Supplementary Information [file 41541_2024_936_MOESM1_ESM.pdf]

<u>Supplementary table and figures</u>

Antibodies	Fluorochrome	Clone	Company	Cat. Number
CD29	**BV786**	**MAR4**	**BD**	**564815**
CD49D	**PE**	**9f10**	**BD**	**560972**
CD45RA	**PEcy7**	**HI100**	**BD**	**560675**
CCR7	**BV510**	**2_L1-A**	**BD**	**566760**
CD19	**AH7**	**SJ25C1**	**BD**	**560177**
CD14	**AH7**	**M5E2**	**BD**	**560270**
CD8	**BB515**	**RPA-T8**	**BD**	**564526**
CD4	**PerCPCy5.5**	**RPA-T4**	**Biolegend**	**300530**
CD3	**APC-R700**	**UCHT1**	**BD**	**659119**
IL-2	**BV605**	**MQ1-17H12**	**Biolegend**	**500332**
IFN-γ	**BV421**	**B27**	**BD**	**562988**
TNF-α	**APC**	**MAb11**	**Biolegend**	**502912**

Supplementary table 1: List of fluorochrome labelled anti-human monoclonal antibodies.

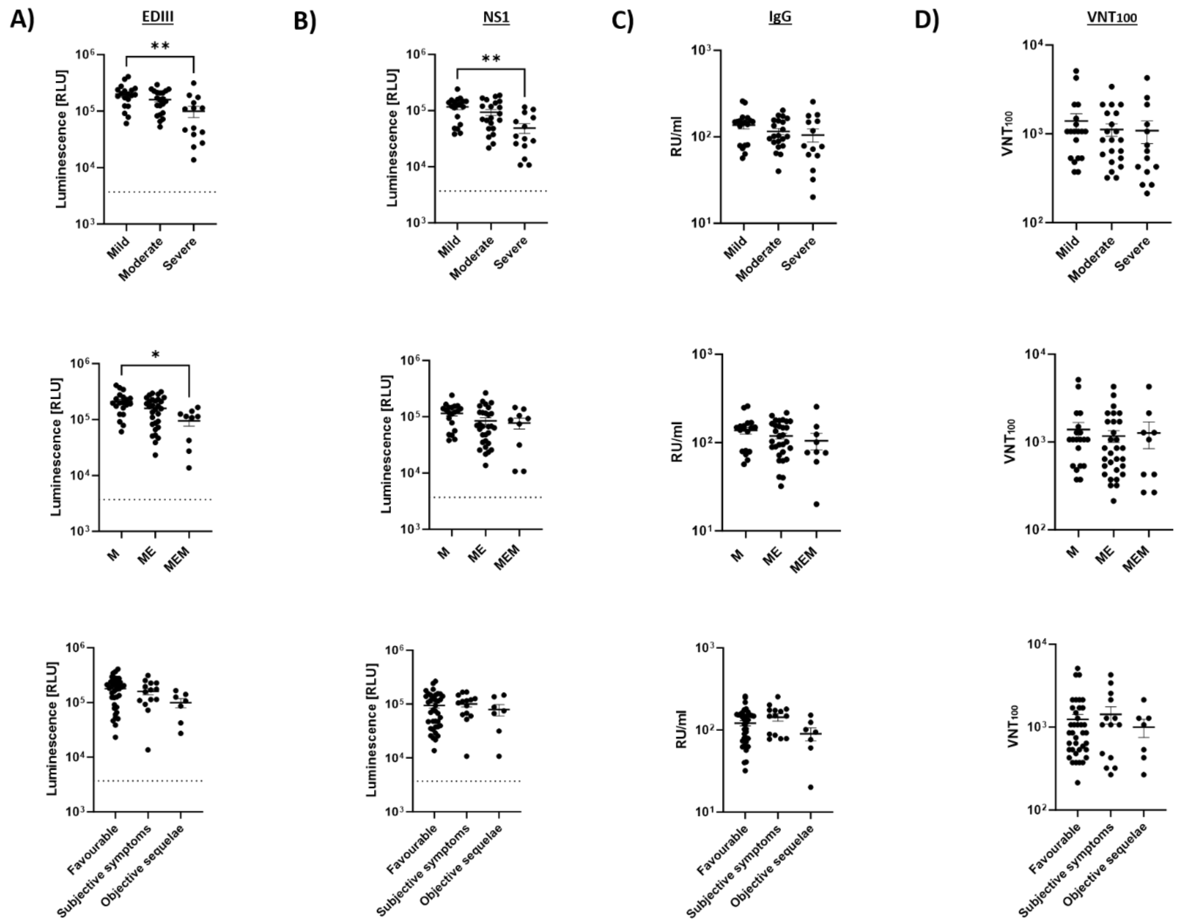

Supplementary figure 1: TBEV EDIII- and NS1- specific antibody titers years after TBE correlate with mild acute disease.

(A - D) (Top panel) TBEV-specific antibody levels in TBE patients who had mild, moderate or severe TBE. **(Middle panel)** IgG, VN, EDIII- and NS1-specific serum antibody titers upon convalescence according to neurological manifestations during hospitalization (M=meningitis, ME=meningoencephalitis, MEM= meningoencephalomyelitis). **(Bottom panel)** analyses of TBEV-specific antibody levels in connection with the long-term clinical outcome of infection. Horizontal lines indicate mean with SEM. Two-way ANOVA with multiple comparison test was performed for comparison of groups. **p<.05, **p<.01*

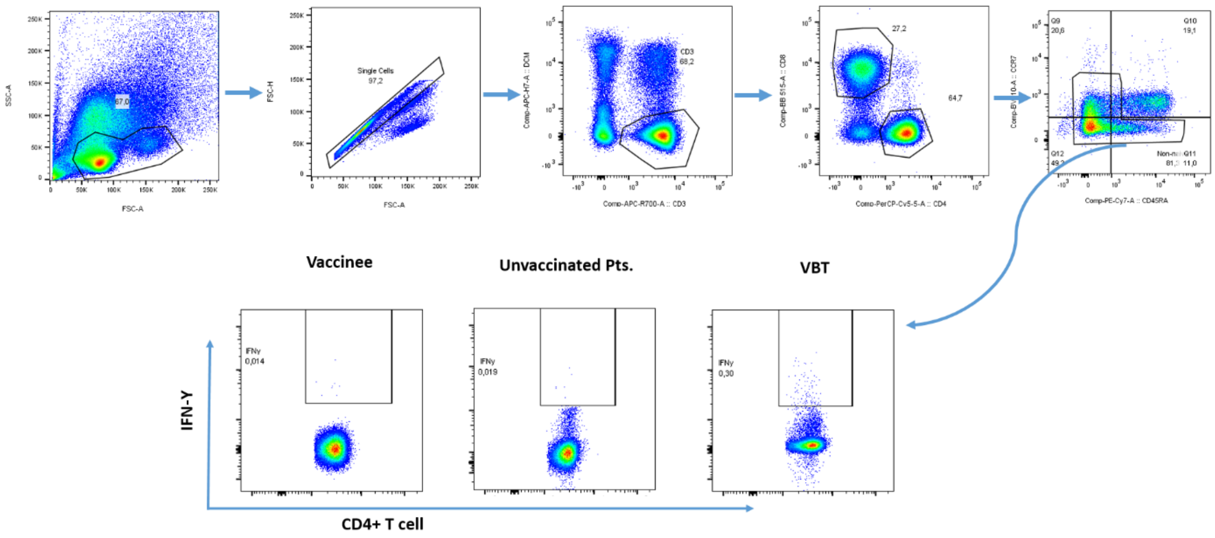

Supplementary figure 2: Representative flow cytometry gating strategy.

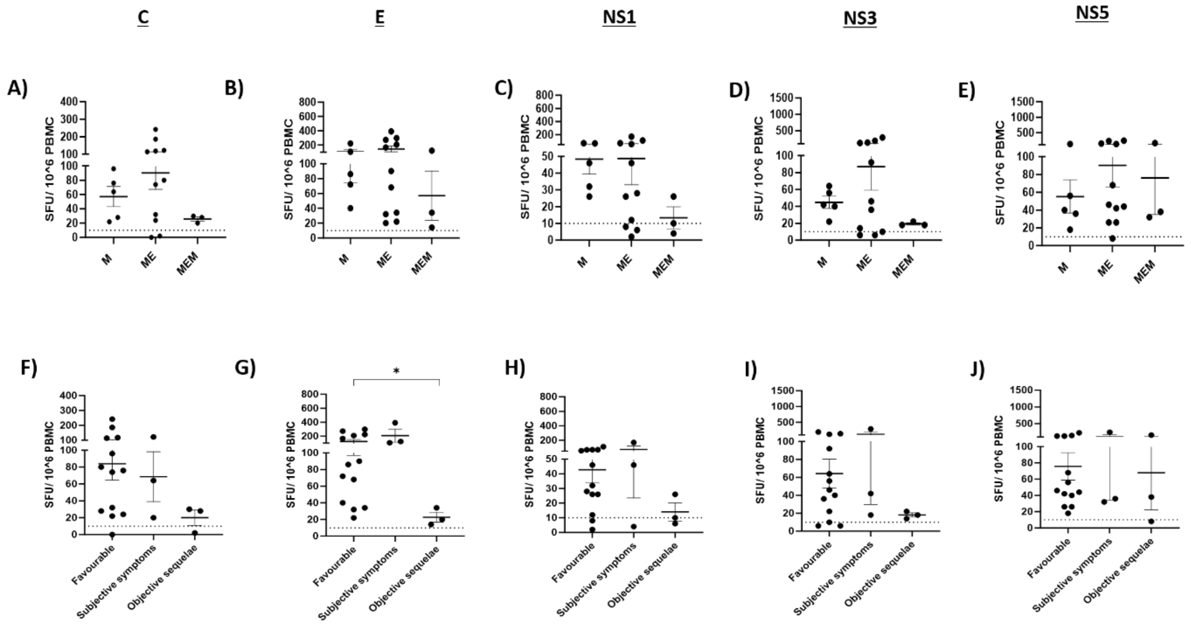

Supplementary figure 3: Analyses of TBEV-specific T cells upon convalescence in relation to disease manifestation during hospitalization and long-term clinical outcome.

(A-E) TBEV-specific T cell IFN-γ responses in unvaccinated patients with the distinct neurological symptoms early after infection (M=meningitis, ME=meningoencephalitis, MEM= meningoencephalomyelitis). **(F -J)** TBEV-specific T cell IFN-γ responses by the time of convalescence and long-term clinical outcomes of infection. Horizontal lines indicate mean

with SEM. Dashed lines indicate cut-off values for positive response. Two-way ANOVA with multiple comparison test was performed for comparison of groups. **p<.05*

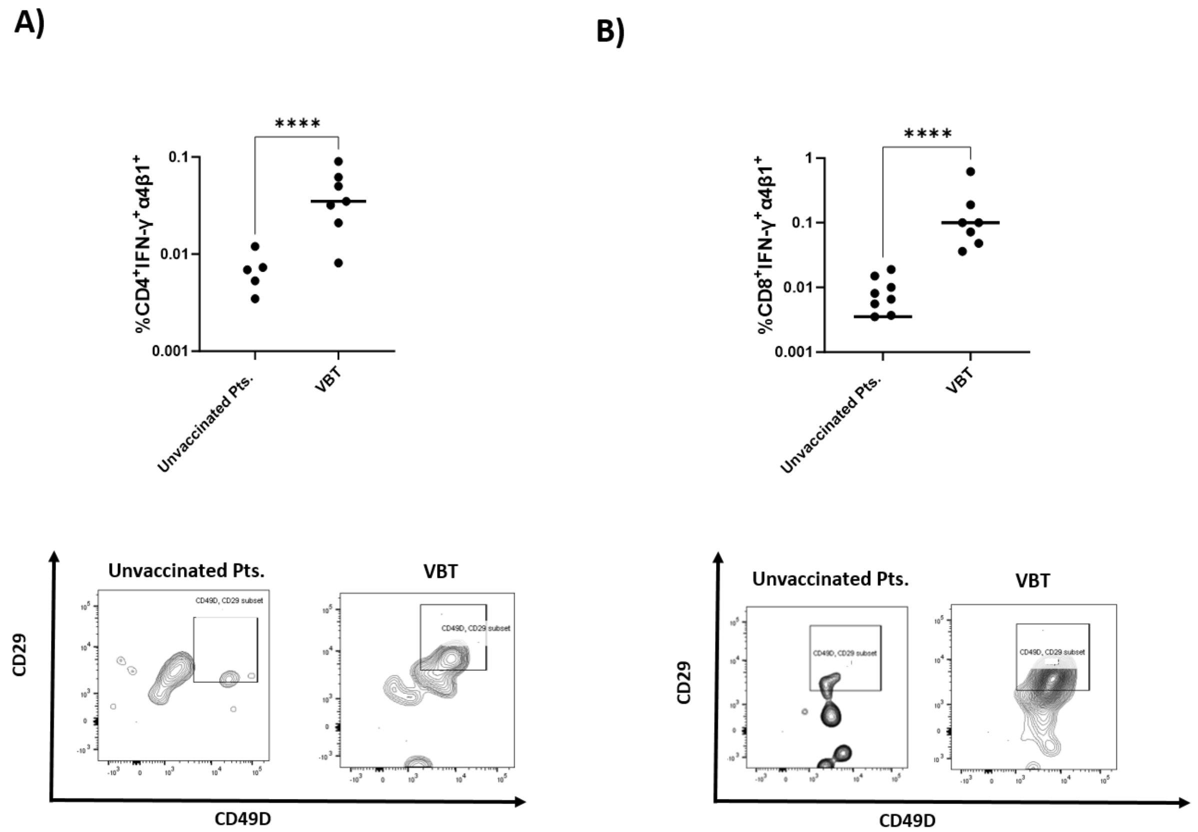

Supplementary figure 4: Expression of α4β1 on TBEV-specific CD4⁺ and CD8⁺ T cells in unvaccinated TBE patients and VBT cases.

Frequency of IFN-γ producing TBEV-specific CD4⁺ **(A)** and CD8⁺ **(B)** T cells that express CD29 and CD49D (alpha4beta1 integrin; α4β1) in unvaccinated patients and VBT cases (top panel). The corresponding representative CD4⁺ **(A)** and CD8⁺ **(B)** T cell FACS plots are depicted (lower panel). Each dot represents single study participant and horizontal lines indicate median values. Man-Whitney test was performed for comparisons of groups. *****p<.0001*

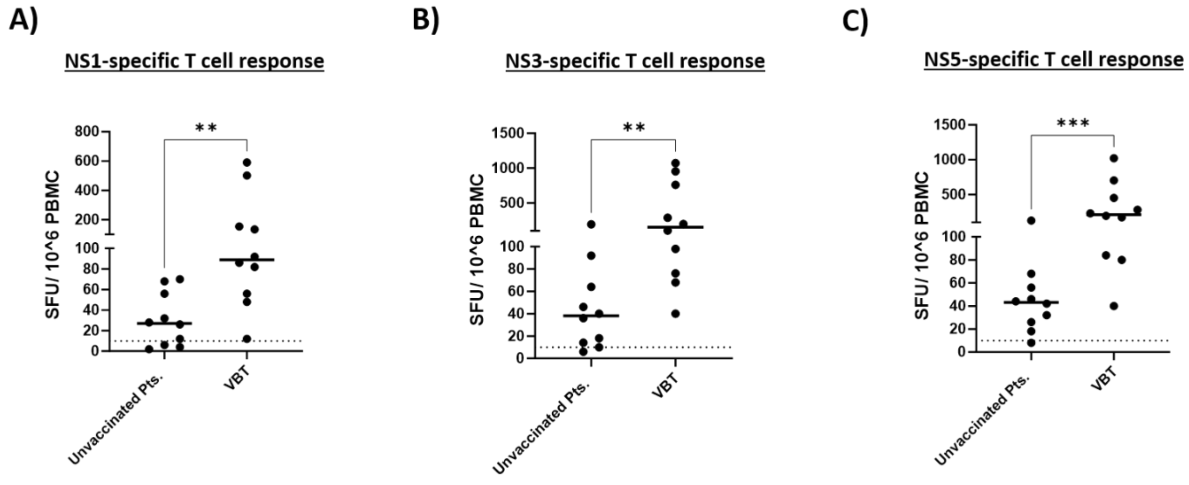

Supplementary figure 5: NS-specific T cell responses in VBT cases and unvaccinated TBE patients in subjects with comparable NS1-specific antibody levels.

(A - C) TBEV-specific IFN-γ spot forming units (SFU) per 1x106 PBMC after stimulation with peptide pools derived from NS TBEV proteins in VBT cases and unvaccinated patients. Ten out of the nine-teen unvaccinated TBE patients with the lowest NS1 antibody levels, comparable to those of the VBT cases, were selected. The sum of individual values obtained with peptide pools per TBEV protein was used to calculate the response to each protein. Each dot represents single study participant and the horizontal lines indicate median values. Dashed lines indicate cut-off values for positive responses. Man-Whitney test was performed for comparisons of groups. **p<.001
